# SUMOylation Inhibition Enhances Protein Transcription under CMV Promoter: A Lesson from a Study with the F508del-CFTR Mutant

**DOI:** 10.3390/ijms25042302

**Published:** 2024-02-15

**Authors:** Christian Borgo, Claudio D’Amore, Valeria Capurro, Valeria Tomati, Nicoletta Pedemonte, Valentina Bosello Travain, Mauro Salvi

**Affiliations:** 1 Department of Biomedical Sciences, University of Padova, 35131 Padova, Italy; christian.borgo@unipd.it (C.B.); claudio_damore@icloud.com (C.D.); 2 UOC Genetica Medica, IRCCS Istituto Giannina Gaslini, Via Gerolamo Gaslini 5, 16147 Genova, Italy; valeriacapurro@gaslini.org (V.C.); valeriatomati@gaslini.org (V.T.); nicolettapedemonte@gaslini.org (N.P.); 3 Department of Molecular Medicine, University of Padova, 35131 Padova, Italy; valentina.bosellotravain@unipd.it

**Keywords:** genetic disease, SUMOylation, drugs, CMV-promoter, ubiquitin, misfolded mutants, proteostasis

## Abstract

Cystic fibrosis (CF) is a genetic disorder caused by mutations in the gene encoding the cystic fibrosis transmembrane conductance regulator (*CFTR*), a selective anion channel expressed in the epithelium of various organs. The most frequent mutation is F508del. This mutation leads to a misfolded CFTR protein quickly degraded via ubiquitination in the endoplasmic reticulum. Although preventing ubiquitination stabilizes the protein, functionality is not restored due to impaired plasma membrane transport. However, inhibiting the ubiquitination process can improve the effectiveness of correctors which act as chemical chaperones, facilitating F508del CFTR trafficking to the plasma membrane. Previous studies indicate a crosstalk between SUMOylation and ubiquitination in the regulation of CFTR. In this study, we investigated the potential of inhibiting SUMOylation to increase the effects of correctors and enhance the rescue of the F508del mutant across various cell models. In the widely used CFBE41o-cell line expressing F508del-CFTR, inhibiting SUMOylation substantially boosted F508del expression, thereby increasing the efficacy of correctors. Interestingly, this outcome did not result from enhanced stability of the mutant channel, but rather from augmented cytomegalovirus (CMV) promoter-mediated gene expression of F508del-CFTR. Notably, CFTR regulated by endogenous promoters in multiple cell lines or patient cells was not influenced by SUMOylation inhibitors.

## 1. Introduction

Cystic fibrosis (CF) is a recessive genetic disease caused by mutations in the gene encoding the cystic fibrosis transmembrane conductance regulator (CFTR), a selective anion channel expressed in the epithelia of various organs. More than 2000 mutations have been identified in the CFTR gene, most of which are thought to be disease-relevant [1,2]. Pathological mutations can result in defects in protein expression, impairment of protein stability, and defects in trafficking/maturation, gating, and conduction [3,4]. The F508del-CFTR mutation (deletion of phenylalanine at position 508) is found in at least one allele in approximately 90% of CF patients worldwide. F508del is the prototype of misfolded mutants, as this single amino acid deletion is responsible for defective folding and processing of CFTR. Consequently, CFTR is unable to reach the plasma membrane and is retained in the endoplasmic reticulum (ER), rapidly ubiquitinated and degraded via the ubiquitin-proteasome system [5]. Moreover, F508del-CFTR has gating defects [6]. Screening large chemical libraries through high-throughput methods revealed small molecules capable of improving the stability and trafficking of F508del-CFTR. These compounds may function as correctors, directly binding to the misfolded protein as chemical chaperones, or alternatively, as proteostasis regulators, influencing cell signaling indirectly. Lumacaftor (VX-809) was identified as a corrector compound that showed promising effects in improving the trafficking of the F508del-CFTR protein. It acts as a pharmacological chaperone inserting into a hydrophobic pocket in the first transmembrane domain (TMD1) linking together four helices that are thermodynamically unstable [7]. Orkambi, a combination of VX-809 and VX-770 (ivacaftor), a potentiator that functions increasing CFTR channel activity [8], is a CF treatment approved by the FDA in 2018. However, this dual-drug combination led to modest clinical improvements in patients [9]. In 2019, a triple combination of two correctors VX-445 (elexacaftor) and VX-661, (tezacaftor) targeting different domains of CFTR with a synergistic effect [10], plus the potentiator ivacaftor has been approved by the FDA with the name of Trikafta [11]. Trikafta has shown remarkable outcomes in treating CF [12]. However, a further improvement in F508del-CFTR rescue is still possible [12,13].

The CFTR protein undergoes various post-translational modifications (PTMs) along with ubiquitination. These PTMs include phosphorylation, SUMOylation, and methylation (for a comprehensive list please refer to the database www.phosphosite.org [14], (accessed on 13 December 2023)) mainly localized in the CFTR regulatory region [15,16,17,18]. Given the importance of PTMs in regulating protein function, stability and localization, researchers have explored the possibility of targeting the enzymes responsible for these modifications to rescue or restore the functionality of mutant CFTR proteins. Numerous phosphorylation sites in CFTR have been identified, with phosphorylation being the most common PTM. Phosphorylation of the channel’s regulatory domain by Protein kinase C (PKC) and Protein kinase A (PKA) activates the channel [15,19]. The most significant and well-characterized phosphorylation sites are those generated by the protein kinase PKA [20,21]. Indirect activation of PKA has been proposed as a promising strategy by Ghigo and colleagues, achieved by slowing down cAMP degradation at the plasma membrane level [22]. The protein kinase CK2 has also been identified as a potential pharmacological target for the rescue of F508del-CFTR. At first, it was suggested that inhibiting CK2 activity might have a beneficial effect on CFTR function. However, further research has revealed that CK2 activity is necessary for the proper functioning of the CFTR channel [16,23], but it is still not known which target sites phosphorylated by CK2 are functionally relevant [24].

As mentioned above, the F508del mutant is the prototype of misfolded mutants that become trapped in the ER and undergo rapid degradation through ubiquitin-dependent proteasomal pathways. Inhibition of protein ubiquitination has long been considered a promising therapeutic approach for CF [15,25]. However, to date, no specific molecule targeting ubiquitination has reached clinical application. Recent studies have highlighted the importance of targeting the early stages of the ubiquitination cascade for effective intervention [26,27,28]. While preventing F508del-CFTR degradation alone is not sufficient to rescue functional channels, these studies demonstrate that inhibiting the early stages of the ubiquitination cascade significantly increases the efficacy of correctors in restoring functional channels [26,27,28]. However, one challenge with this approach is the potential toxicity associated with global inhibition of protein ubiquitination. In an attempt to overcome this issue, and taking into account the observation that certain lysine residues in CFTR can undergo both ubiquitination and methylation, we previously explored the hypothesis that preventing demethylation might be an effective strategy to block ubiquitination and degradation of the CFTR channel. This study identified the lysine demethylases KDM2A and KDM3B as possible targets for increasing the stability of the F508del protein [29].

A crosstalk between ubiquitination and SUMOylation has also been suggested as an important mechanism to control F508delCFTR degradation [30,31]. In particular, it has been shown that SUMOylation is required for RNF4-mediated ubiquitination and subsequent degradation of F508del-CFTR, whereas it has little effect on the wild-type form [32]. The process involves HSP27, a small chaperone, recognizing the misfolded F508del mutant, which then facilitates the recruitment of the E2 enzyme UBC9. UBC9 triggers polySUMOylation (SUMO-2/3) of the CFTR channel. The resulting polySUMO chain serves as a signal for the SUMO-targeted ubiquitin ligase RNF4, which identifies the polySUMO chain and initiates protein polyubiquitination, causing degradation of the F508del mutant [32].

Additionally, it was discovered that the E3 ligase PIAS4 also plays a role in the SUMOylation of F508del CFTR. This enzyme potentially affects the same lysine residues in the NBD1 domain but in a contrasting manner. PIAS4 attaches SUMO-1 to the F508del mutant, which is a distinct paralog of SUMO compared to the polySUMO chain mediated by UBC9 (SUMO-2/3). This suggests that PIAS4-mediated monoSUMOylation competes with UBC9-mediated polySUMOylation. SUMOylation of F508del-CFTR by PIAS4 (mono-SUMO-1) prevents the formation of the polySUMOylation chain (SUMO-2/3) and the resulting RNF4-dependent ubiquitination, thereby enhancing the stability of the F508del [33].

Based on these findings, we hypothesized that blocking the entire SUMOylation process, both mono- and poly-SUMOylation, may prevent the specific degradation of F508del-CFTR, which is RNF4-dependent, thereby increasing the cellular amount of mutant CFTR. Consequently, this could elevate the cellular level of mutant CFTR and potentially enhance channel rescue when used in conjunction with correctors.

Most preclinical research on CF is conducted using immortalized cell lines. Various immortalized cell lines are employed in CF research, with their widespread use attributed to advantages such as easy culturing and rapid expansion, facilitating high-throughput drug screening, and easy transfectable for experiments of downregulation/overexpression [34]. Fischer rat thyroid cells expressing F508del-CFTR have played a crucial role in drug discovery, notably contributing to the identification of the CFTR modulator ivacaftor [34].

The most commonly used immortalized lines include CF (CFBE41o-) and wild-type (16HBE14o-) human bronchial epithelial cells [35]. Despite CFBE41o- being derived from a CF patient homozygous for the F508del-CFTR mutation, the endogenous *CFTR* gene expression is low [36]. Consequently, for functional studies, CFBE41o- cell lines are typically stably transfected to express a sufficient amount of F508del-CFTR.

In our study, CFBE41o- cells transfected with F508del-CFTR under the control of a cytomegalovirus (CMV) promoter [37] were employed. Additionally, we utilized the same cells stably cotransfected with the halide-sensitive yellow fluorescent protein (HS-YFP) for CFTR activity assays [38]. The CMV promoter stands out as the most widely used promoter in many eukaryotic expression vectors for its high transcription level.

The SUMOylation machinery, like ubiquitination, consists of a cascade of three enzymes: two E1 enzymes (SAE1 and SAE2), a single E2 enzyme, UBC9, which can directly transfer SUMO to targets without the aid of E3 ligases, and less than a dozen E3 enzymes [39].

In this study, we explored various techniques to prevent SUMOylation, including the use of small molecule inhibitors targeting E1 and E2 enzymes, and transient downregulation of UBC9 using RNA interference.

These approaches resulted in increased levels of the F508del mutant and improved the effects of correctors on restoring the mutant CFTR channel in CFBE41o- cells expressing F508del-CFTR. Nevertheless, contrary to expectations, the increase in F508del protein levels following SUMOylation inhibition was not due to variations in protein stability, but instead to an increase in protein transcription. Notably, this effect was solely observed with the artificial promoters used in the study and did not affect the endogenous CFTR promoter.

These results emphasize the importance of being cautious when interpreting the impact of manipulating SUMO pathways on exogenously expressed proteins governed by promoters like CMV, which is frequently utilized for recombinant protein expression.

## 2. Results

**Figure 1:**
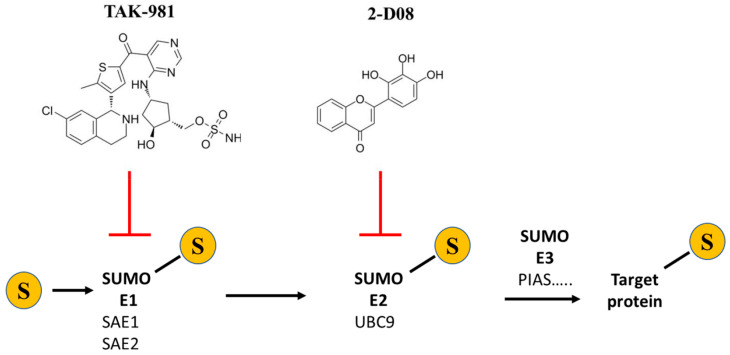
Structure and targets of SUMOylation inhibitors TAK-981 and 2-D08.

In this study, we used two different chemical inhibitors to prevent in-cell protein SUMOylation: the UBC-9 inhibitor 2-D08 [40] and the SAE inhibitor TAK-981 (subasumstat) [41]. These inhibitors act on the E2 (Ubc-9) and E1 (SAE) enzymes, respectively, preventing the entire process of SUMOylation. Figure 1 displays the structures of the compounds as well as their targets in the SUMOylation pathway.

**Figure 2:**
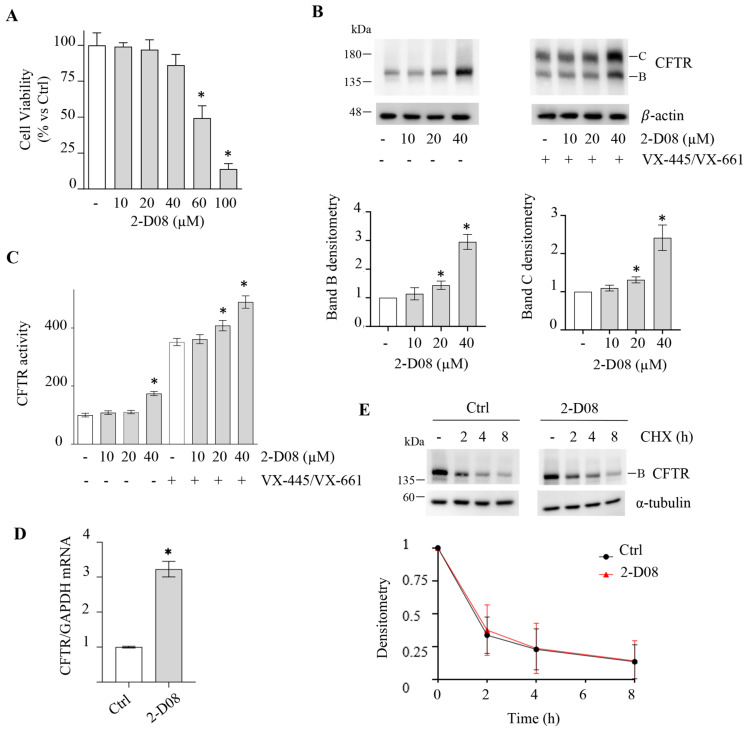
Effect of 2-D08 on F508del-CFTR expression and activity in CFBE41o- cells. (**A**) The viability of F508del-CFTR expressing CFBE41o- cells was assessed by the MTT assay after 24 h treatment with DMSO (Ctrl, -) or increasing concentrations of 2-D08 and expressed as a percentage of Ctrl (means ± SD values, n = 3; * *p* < 0.05 vs. Ctrl). (**B**) Immunoblot analysis of F508del-CFTR in whole lysates from F508del-CFTR expressing CFBE41o- cells treated with DMSO (−) or increasing concentrations of 2-D08 with (+) or without (−) the double corrector treatment VX-445/VX-661 (3 μM VX-445 + 10 μM VX-661) for 24 h. β-actin was used as a loading control. The lower panels show the densitometric quantification of the immunostained F508del-CFTR band B and band C, normalized to β-actin amount, in the experiment detailed in the upper panels. The values for CFTR band B are expressed as a percentage of DMSO-treated cells (means ± SD values, n = 3; * *p* < 0.05 vs. DMSO-treated cells) and for CFTR band C are expressed as a percentage of cells treated only with VX-445/VX-661 (means ± SD values, n = 3; * *p* < 0.05 vs. VX-445/VX-661-treated cells). (**C**) HS-YFP assay in F508del-CFTR expressing CFBE41o- cells treated as indicated in panel B. The CFTR activity is expressed as a percentage of control (−) (means ± SD values, n = 8; * *p* < 0.05 vs. “-”). (**D**) *CFTR* mRNA level determined by quantitative real-time PCR in F508del-CFTR expressing CFBE41o- cells were treated with DMSO (Ctrl) or 2-D08 (40 µM) for 24 h. *CFTR* mRNA expression was normalized to *GAPDH* and reported relative to its expression in Ctrl cells that was arbitrarily set to 1 (means ± SD values, n = 3, * *p* < 0.05 vs. Ctrl). (**E**) F508del-CFTR expressing CFBE41o- cells were treated with DMSO (Ctrl) or 2-D08 (40 µM) for 24 h. Subsequently, protein synthesis was inhibited by adding 100 µg/mL cycloheximide (CHX), and cells were harvested at the indicated times. Protein lysates were analyzed by western blot with an anti-CFTR antibody. α-tubulin was used as a loading control. Lower panel represents the densitometric quantification of the immunostained bands of F508del-CFTR band B firstly normalized to α-tubulin amount and subsequently normalized by the value at time = 0 in the experiment detailed in the upper panels (means ± SD values, n = 3; * *p* < 0.05 vs. the value of the same time-point of Ctrl).

To evaluate the effect of the inhibitors on F508del protein expression, we treated CFBE41o- cells harboring the homozygous deletion of Phe-508 in CFTR and expressing F508del-CFTR with increasing doses of 2-D08 (ranging from 10 to 40 μM). The selected concentration range of the inhibitor did not affect cell proliferation after 24 h of treatment (as shown in Figure 2A) (the 48 h treatment is shown in Figure S1).

The F508del-CFTR protein expression was assessed by Western blotting. Western blot analysis of cell lysates using CFTR antibodies is used not only to quantify protein expression but also to monitor channel maturation. Wild-type (WT) CFTR typically appears as a double band: a distinct upper band at about 180 kDa, termed band C, and a smaller lower band at about 160 kDa, termed band B. Band C represents the fully glycosylated mature form, which resides in the plasma membrane, whereas band B represents the partially glycosylated immature form, which resides in the ER. In the case of F508del-CFTR, only band B is observed due to the phenylalanine deletion, which prevents its maturation to band C. Addition of correctors to cells expressing F508del-CFTR induces the appearance of band C as a result of a partial rescue of the channel [42].

Cell treatments with 2-D08 increase the amount of band B in F508del-CFTR (Figure 2B), indicating that the inhibitor increases the amount of immature form of the mutant protein. However, the lack of CFTR band C suggests the absence of F508del-CFTR maturation (Figure 2B).

Although 2-D08 does not induce F508del-CFTR maturation, we wondered whether Vertex Pharmaceutical’s VX-445 and VX-661 compounds could correct the raised level of F508del-CFTR band B triggered by the SUMOylation inhibitor 2-D08. Both correctors, VX-445 and VX-661, are components of Trikafta, the current therapy for individuals with at least one copy of F508del-CFTR and we wondered if 2-D08 cotreatment could enhance the efficacy of these molecules. CFBE41o- cells expressing F508del-CFTR were treated with VX-445 and VX-661 (double corrector treatment) in combination with or without 2-D08. As expected, double corrector treatment alone induced maturation of F508del-CFTR, as indicated by the presence of band C (fully glycosylated mature form). However, when VX-445/VX-661 is combined with 2-D08, the amount of band C is further increased (Figure 2B). Importantly, the ratio of band C to band B does not increase in the presence of 2-D08 compared to VX-445/VX-661 alone, indicating that the effect of the inhibitor is solely to increase the amount of F508del-CFTR in the ER available for correction (Figure 2B).

To assess the functional rescue of F508del-CFTR after VX-445/VX-661 and 2-D08 treatments, the CFTR-dependent iodide transport was measured by fluorescent quenching of stably expressed halide-sensitive YFP in CFBE41o- cells. The CFTR channel demonstrates permeability not only to chloride and bicarbonate, but also to halides such as iodide, which are not naturally present in the intra- or extra-cellular space. Using halides facilitates the specific measurement of CFTR permeability, as other anion cotransporters lack permeability to halides. In this assay, cells co-expressing HS-YFP and F508del-CFTR were subjected to treatment with correctors, with or without 2-D08. After 24 h-treatment cells were incubated in an iodide-rich buffer and CFTR activity was assessed by measuring the rate of YFP fluorescence quenching. This quenching is a result of CFTR-dependent iodide uptake at the plasma membrane following the activation of the channel [43]. The results confirmed that 2-D08 significantly improved the functional rescue of F508del-CFTR induced by the correctors VX-445 and VX-661 (Figure 2C). However, even when used alone, a higher dose of 2-D08 produced a small increase in F508del-CFTR function (Figure 2C), even though 2-D08 alone is unable to induce channel maturation (Figure 2B). This phenomenon could be explained by the movement of a small fraction of F508del-CFTR (immature form) through the unconventional pathway to the plasma membrane, which depends on the channel amount found in the ER. Indeed, in untreated CFBE41o- cells expressing F508del-CFTR, a channel activity, which is suppressed by siRNA downregulation of F508del-CFTR, is measurable by the YFP assay [29].

An increase in protein level may be due to an increase in the amount of mRNA or an increase in protein stability. To assess the effect of 2-D08 treatment on F508del-CFTR gene transcription, qRT-PCR was executed. The results showed that treatment of CFBE41o- cells with 40 μM 2-D08 induced an approximately threefold increase in F508del-CFTR mRNA levels compared to the control (Figure 2D).

To evaluate the effect of 2-D08 treatment on F508del protein stability, a cycloheximide (CHX) assay was performed. The cycloheximide assay is a common method used to assess the stability of a protein in cells. Cycloheximide is a translation inhibitor that binds to the ribosome, preventing new protein synthesis. This allows for the monitoring of the decay of pre-existing proteins over time. CFBE41o- cells were treated with or without 40 μM 2-D08. After 24 h of treatment, cells were incubated with CHX to block protein synthesis and samples were collected at different time points for analysis. The results shown in Figure 2E indicate that F508del-CFTR stability is not significantly impacted by 2-D08 treatment.

These results suggest that the increase in F508del-CFTR protein level induced by 2-D08 treatment is primarily mediated by an increase in its mRNA level rather than changes in protein stability.

**Figure 3:**
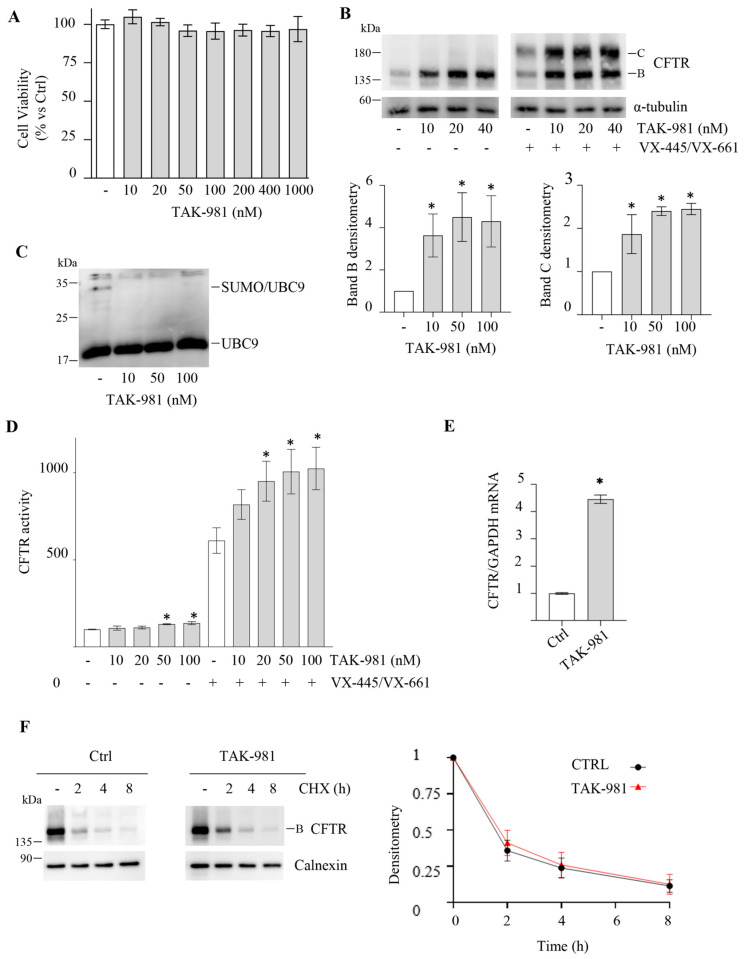
Effect of TAK-981 on F508del-CFTR expression and activity in CFBE41o- cells. (**A**) The viability of F508del-CFTR expressing CFBE41o- cells was assessed by the MTT assay after 24 h treatment with DMSO (Ctrl, -) or increasing concentrations of TAK-981 and expressed as a percentage of Ctrl (means ± SD values, n = 3; * *p* < 0.05 vs. Ctrl). (**B**) Immunoblot analysis of F508del-CFTR in whole lysates from F508del-CFTR expressing CFBE41o- cells treated with DMSO (−) or increasing concentrations of TAK-981 in combination (+) or not (−) with the VX-445/VX-661 treatment (3 μM VX-445 + 10 μM VX-661) for 24 h. α-tubulin was used as a loading control. The lower panels show the densitometric quantification of the immunostained F508del-CFTR band B and band C, normalized to α-tubulin amount, in the experiment detailed in the upper panels. The values for CFTR band B are expressed as a percentage of DMSO-treated cells (means ± SD values, n = 3; * *p* < 0.05 vs. DMSO-treated cells) and for CFTR band C are expressed as a percentage of cells treated only with VX-445/VX-661 (means ± SD values, n = 3; * *p* < 0.05 vs. VX-445/VX-661-treated cells). (**C**) Cellular lysates were analyzed by western blot with anti-UBC9 antibody. Slower mobility of SUMO–UBC9 thioester complexes (SUMO/UBC9) was detected in the absence of reducing agent (-DTT). (**D**) HS-YFP assay in F508del-CFTR expressing CFBE41o- cells treated as indicated in panel B. The CFTR activity is expressed as a percentage of control (−) (means ± SD values, n = 8; * *p* < 0.05 vs. “-”). (**E**) *CFTR* mRNA level determined by quantitative real-time PCR in F508del-CFTR expressing CFBE41o- cells were treated with DMSO (Ctrl) or TAK-981 (100 nM) for 24 h. *CFTR* mRNA expression was normalized to *GAPDH* and reported relative to its expression in Ctrl cells that was arbitrarily set to 1 (means ± SD values, n = 3, * *p* < 0.05 vs. Ctrl). (**F**) F508del-CFTR expressing CFBE41o- cells were treated with DMSO (Ctrl) or TAK-981 (100 nM) for 24 h. Subsequently, protein synthesis was inhibited by adding 100 µg/mL cycloheximide (CHX), and cells were harvested at the indicated times. Protein lysates were analyzed by western blot with an anti-CFTR antibody. Calnexin was used as a loading control. Lower panel represents the densitometric quantification of the immunostained bands of F508del-CFTR band B firstly normalized to calnexin amount and subsequently normalized by the value at time = 0 in the experiment detailed in the upper panels (means ± SD values, n = 3; * *p* < 0.05 vs. the value of the same time-point of Ctrl).

To support the results obtained with 2-D08, we inhibited the SUMOylation process by using a different molecule, the recently discovered highly specific SAE (E1) inhibitor, TAK-981. TAK-981, as well as 2-D08 prevent the entire SUMOylation cascade (see Figure 1). CFBE41o- cells expressing F508del-CFTR were treated with increasing doses of TAK-981 (ranging from 10 to 100 nM). TAK-981 does not affect the proliferation of CFBE41o- cells, even at high concentrations (up to 1 µM) (Figure 3A) (the 48 h treatment is shown in Figure S1).

Treatment of CFBE41o- cells with TAK-981 resulted in a marked increase in F508del-CFTR band B expression through Western blotting, with no effect on band C expression (Figure 3B), similar to 2-D08. TAK-981 also increases the protein amount of wild-type CFTR transiently expressed in CFBE41o- cells (Figure S2). In addition, TAK-981 was able to potentiate the rescue of F508del-CFTR at the plasma membrane induced by VX-445 and VX-661, as demonstrated by increased band C expression in Western blotting (Figure 3B). Non-reducing SDS-PAGE was carried out to confirm the inhibition of the SUMOylation process by TAK-981. UBC9 can form thioester bonds with SUMO (UBC9-SUMO), which are sensitive to reducing agents. Under non-reducing conditions, UBC9-SUMO thioester species show a slower migration rate compared to UBC9 alone. Consistent with the inhibition of the SUMOylation cascade (E1 inhibition), TAK-981 treatment resulted in a decrease in the level of DTT-sensitive UBC9-SUMO thioesters (Figure 3C).

The functional assay (YFP) confirmed that TAK-981 was able to potentiate the functional rescue effects of VX-445/VX-661 treatment, as demonstrated by increased channel activity (Figure 3D). Likely 2-D08, TAK-981 alone induces a slight increase in channel activity (Figure 3D), probably due to the unconventional trafficking pathway, as it alone is unable to induce the appearance of band C.

To further understand the underlying mechanisms, we examined the effect of TAK-981 on F508del-CFTR gene transcription and protein stability. The results showed that treatment with 100 nM TAK-981 resulted in a 4-fold increase in F508del-CFTR mRNA levels, supporting the notion that the compound enhances gene transcription (Figure 3E). However, similar to 2-D08, TAK-981 had no significant effect on F508del-CFTR protein stability as assessed by the CHX assay (Figure 3F).

**Figure 4:**
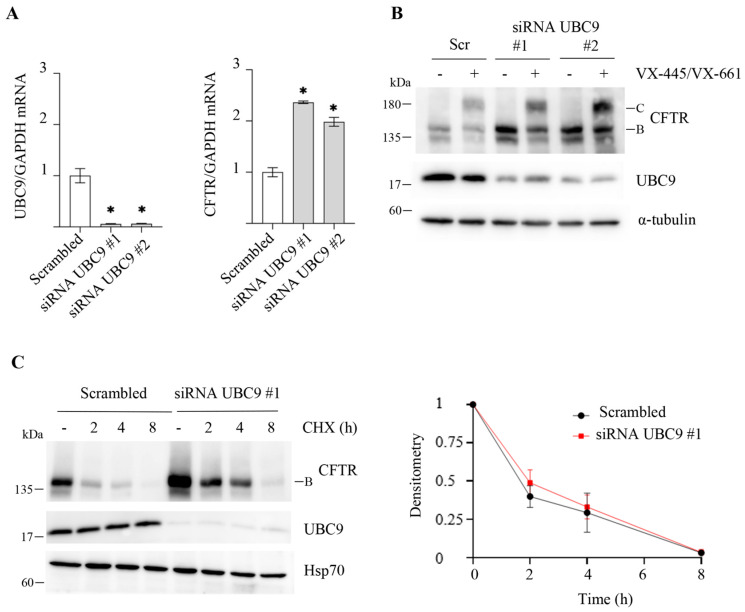
Effect of UBC-9 downregulation on F508del-CFTR expression and activity in CFBE41o- cells. F508del-CFTR expressing CFBE41o- cells were transfected with non-specific siRNA (Scrambled, Scr), or two different UBC9-specific siRNAs (#1, #2) for 72 h. (**A**) *UBC9* and *CFTR* mRNA levels were determined by quantitative real-time PCR. *UBC9* and *CFTR* mRNA expression was normalized to *GAPDH* and reported relative to its expression in cells treated with scrambled siRNA that was arbitrarily set to 1 (means ± SD values, n = 3, * *p* < 0.05 vs. scrambled siRNA-treated cells). (**B**) 48 h after siRNA transfection, cells were treated for 24 h with (+) or without (−) a combination of 3 μM VX-445 + 10 μM VX-661 (VX-445/VX-661). Lysate proteins were analyzed by western blot with the indicated antibodies. α-tubulin was used as loading control. (**C**) After 72 h of siRNA treatments, protein synthesis was inhibited by adding 100 µg/mL cycloheximide (CHX), and cells were harvested at the indicated times. Protein lysates were analyzed by western blot with an anti-CFTR antibody. Hsp70 was used as a loading control. Lower panel represents the densitometric quantification of the immunostained bands of F508del-CFTR band B firstly normalized to Hsp70 amount and subsequently normalized by the value at time = 0 in the experiment detailed in the upper panels (means ± SD values, n = 3; * *p* < 0.05 vs. the value of the same time-point of cells treated with scrambled siRNA).

We wondered whether the effects of the SUMO pathway inhibitors were due to some off-target effects shared by both molecules. Using a different approach, we performed siRNA-mediated downregulation of UBC9 in CFBE41o- cells. CFBE41o- cells were treated with siRNAs targeting two different sequences in the UBC9 mRNA or with scrambled siRNAs for 72 h. Both UBC9 siRNAs strongly reduced UBC9 mRNA levels (Figure 4A) and protein expression (Figure 4B). Similar to the effect of SUMO inhibitors, UBC9 downregulation leads to an increase in F508del-CFTR band B expression without affecting band C (Figure 4B), which correlates with an increase in F508del-CFTR mRNA levels (Figure 4A), leaving protein stability unaffected (Figure 4C). These results provide additional evidence to support the notion that the effects observed with 2-D08 and TAK-981 result from inhibiting the SUMOylation pathway, rather than from off-target effects shared by both inhibitors.

Our results show that in CFBE41o- cells expressing F508del-CFTR, inhibition of the SUMOylation pathway increases the protein expression of F508del-CFTR as a consequence of transcriptional stimulation.

SUMO modification affects many biological pathways but plays a particularly important role in regulating transcription. Indeed, a large number of transcription factors are SUMOylated and a large body of evidence highlights its role in the negative regulation of transcription [44]. Therefore, our results are consistent with a transcriptional repressive role for SUMO modification of CFTR expression in our cell model.

**Figure 5:**
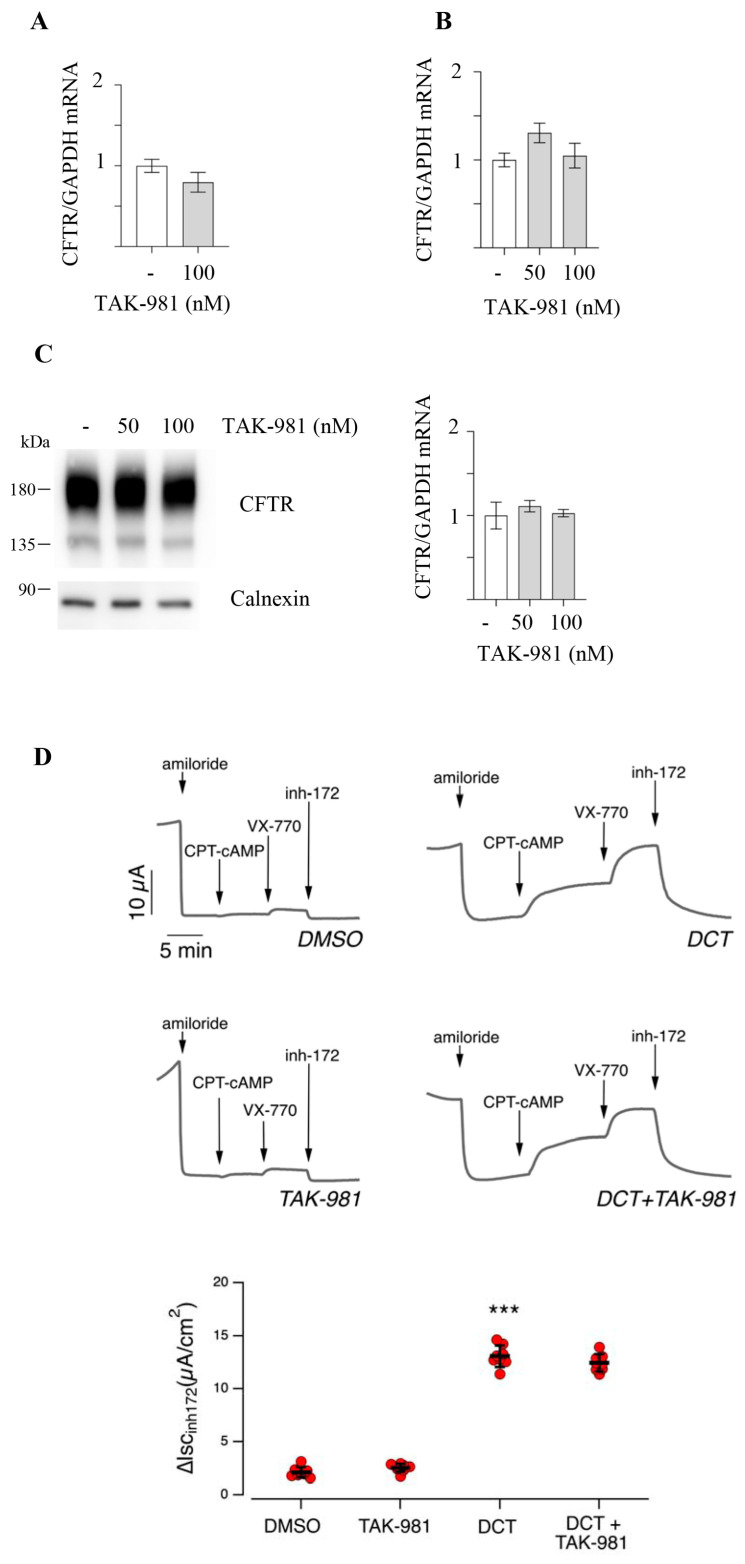
Effect of TAK-981 on endogenous *CFTR* promotors. (**A**) CFBE41o- cells not expressing F508del-CFTR were treated with DMSO (−) or with 100 nM TAK-981. *CFTR* mRNA levels were determined by quantitative real-time PCR. *CFTR* mRNA expression was normalized to *GAPDH* and reported relative to its expression in DMSO-treated cells that was arbitrarily set to 1 (means ± SD values, n = 3). (**B**) 16HBE14o- cells were treated with DMSO (−) or with 50 or 100 nM TAK-981. *CFTR* mRNA levels were determined by quantitative real-time PCR. *CFTR* mRNA expression was normalized to GAPDH and reported relative to its expression in DMSO (−) cells that was arbitrarily set to 1 (means ± SD values, n = 3). (**C**) Calu-3 cells were treated with DMSO (−) or with 50 or 100 nM TAK-981. On the left, immunoblotting analysis of CFTR expression. Calnexin is the loading control. On the right, *CFTR* mRNA levels are determined by quantitative real-time PCR. *CFTR* mRNA expression was normalized to GAPDH and reported relative to its expression in Ctrl cells that was arbitrarily set to 1 (means ± SD values, n = 3). (**D**) Functional evaluation of VX-445/VX-661 and TAK-981 alone or in co-treatment on F508del/F508del human bronchial epithelial cells. F508del/F508del human bronchial epithelial cells were treated with the vehicle alone (DMSO) or TAK-981 (100 nM), or VX-445/ VX-661 (3 µM/10 µM; DCT), or VX-445/VX-661 plus TAK-981 (DCT + TAK-981), with the short-circuit current technique. Data reported are the amplitude of the current blocked by 10 µM inh-172. Symbols indicate statistical significance of treatment vs control (DMSO-treated): *** *p* < 0.001.

Is inhibition of SUMOylation a potential therapeutic target in CF? In our cellular model, F508del-CFTR is ectopically expressed in CFBE41o- cells under a CMV promoter, so the effect of SUMOylation inhibition on F508del-CFTR gene transcription could not be reproduced with endogenous promoters. Therefore, we tested the efficacy of TAK-981 in different cell models expressing endogenous CFTR: CFBE41o- cells expressing endogenous F508del-CFTR, 16HBE14o- and Calu-3 expressing endogenous WT-CFTR. Figure 5A–C shows that TAK-981 does not induce an increase in CFTR mRNA or protein levels in any of the three cellular models. Again, we tested the effect of TAK-981 alone or in combination with correctors in primary epithelia from a patient homozygous for the F508del mutation (Figure 5D). Air-liquid interface (ALI) differentiated airway epithelial cell cultures, as employed in this study, are considered a golden standard for evaluating patients’ responses to pharmacological treatments in preclinical settings [45]. The compound was ineffective both alone and in combination with CFTR correctors (Figure 5D).

## 3. Discussion

The deletion of F508 in CFTR affects protein stability, trafficking, and activity. We and others have previously shown that preventing protein degradation by targeting the E1 enzyme in the ubiquitination pathway is a useful strategy in combination therapy with Vertex Pharmaceuticals’ correctors to improve functional recovery of the channel [26,27]. However, targeting E1 means preventing the entire ubiquitylation process with potential adverse effects. Therefore, we investigated the inhibition of the SUMO pathway as an alternative way to increase protein stability. Indeed, as discussed above, crosstalk between SUMOylation and ubiquitination controls the stability of F508del-CFTR [32,33].

The results presented here show that targeting the SUMOylation pathway is indeed an efficient way to increase the expression of F508del-CFTR stably transfected in CFBE41o- cells. However, the mechanism of action is different from what was expected. Preventing protein SUMOylation does not increase the amount of F508del-CFTR by increasing the stability of the mutant channel but by increasing gene transcription. Since F508del-CFTR cDNA was stably transfected into CFBE41o- under the control of a CMV promoter, we wondered what the effect of SUMOylation inhibition would be on CFTR under the control of the endogenous promoter. Using three different cell lines and primary human bronchial cells from a F508del homozygous patient, we show that SUMOylation inhibition does not affect channel transcription and expression. These results suggest that global inhibition of the SUMOylation pathway is not a therapeutic option for the functional rescue of F508del CFTR.

Our data suggest that SUMOylation affects the transcription of stably transfected F508del-CFTR by regulating the activity of its CMV promoter. This is supported by the fact that TAK-981 also increases the protein amount of F508del-CFTR or wild-type CFTR, which was transiently expressed in CFBE41o- cells using a different plasmid but with the same CMV promoter (see Figure S2). Additionally, this effect extends to other cell types; for instance, TAK-981 augments the protein levels of F508del-CFTR transiently expressed in HEK293 cells with a CMV promoter, as shown in Figure S3A. Similarly, it affects a different protein (CK2α) ectopically expressed through a plasmid under the CMV promoter, without affecting the endogenously expressed one, as demonstrated in Figure S3B.

It is possible that SUMOylation affects the CMV promoter through the regulation of certain transcription factors (TF). While, to our knowledge, there is no literature describing this effect, many transcription factors have been found to be SUMOylated, and SUMOylation is generally associated with reduced or repressed transcription. SUMOylation can impede transcription by recruiting corepressor complexes or disrupting transcription-promoting modifications. Recent studies emphasize that TF SUMOylation primarily alters TF levels at chromatin binding sites, either directly affecting DNA binding or indirectly regulating TF abundance and localization [44].

Our results, although negative, are important because the CMV promoter is the most commonly used promoter for ectopic expression of proteins. Therefore, great care should be taken when interfering with the SUMOylation pathway in cell models with proteins expressed by gene transcription under the CMV promoter.

Our results highlight the importance of evaluating potential therapeutic strategies in relevant physiological contexts, such as primary patient cells, to better understand their efficacy. It also emphasizes the need for careful interpretation of results obtained from ectopic expression systems, as they may not always reflect the behavior of the protein in its native cellular context.

## 4. Materials and Methods

### 4.1. Materials

VX-445, VX-661, TAK-981, and 2-D08 were purchased from MedChemExpress. Anti-CFTR antibodies (#596) were obtained from the Cystic Fibrosis Foundation Therapeutics. Anti-α-tubulin (T5168), and anti-β-actin (A5441) antibodies were purchased from Merck (Darmstadt, Germany). Anti-Calnexin (sc-46669), anti-Hsp70 (sc-32239), anti-UBC9 (sc-271057), and anti-GAPDH (sc-47724) antibodies were from Santa Cruz Biotechnology (Delaware, USA). Anti-CK2α/α′ antibody (MCA3031Z) was from Bio-Rad Laboratories (Hercules, CA, USA). HRP-conjugated anti-mouse and anti-rabbit secondary antibodies were from PerkinElmer (Waltham, MA, USA). siRNA oligos were obtained from Thermofisher (Waltham, MA, USA). The pcDNA3.1 encoding F508del- or WT CFTR vector, a generous gift from Prof. M. Benharouga (University of Grenoble, Grenoble, France), is described in [46]. FLAG-CK2α in pcDNA3.1 plasmid (under the CMV promoter) was purchased from GenScript (Piscataway, NJ, USA).

### 4.2. Cell Culture

CFBE41o- expressing an endogenous level of F508del-CFTR or stably expressing F508del-CFTR [37] and the halide-sensitive yellow fluorescent protein (HS-YFP) YFP-H148Q/I152L [38] were cultured in MEM cell culture medium supplemented with 10% FBS, 2 mM L-glutamine, and antibiotics and maintained at 37 °C in a humidified atmosphere of 5% CO_2_. HEK293T was cultured in DMEM cell culture medium supplemented with 10% FBS, 2 mM L-glutamine and antibiotics and maintained at 37 °C in a humidified atmosphere of 5% CO_2_.

Isolation, culture, and differentiation of primary airway epithelial cells were performed as previously described [26] with some modifications. Bronchial epithelial cells were obtained from mainstem human bronchi derived from CF individuals undergoing lung transplants. Epithelial cells were detached by overnight treatment of bronchi with protease XIV. For the present study, cells were obtained from one F508del homozygous CF patient (HBE93). Airway epithelial cells were cultured in a serum-free medium (LHC9 mixed with RPMI 1640, 1:1) containing various hormones and supplements, which favors cell number expansion. The culture medium contained in the first days a mixture of different antibiotics (including colistin, piperacillin, and tazobactam) to eradicate bacterial contamination.

To obtain differentiated airway epithelia, bronchial cells were seeded at a high density (500.000 cells/cm^2^) on porous membranes (Snapwell inserts, Corning, Corning, NY, USA, code 3801). After 24 h, the serum-free medium was removed from both sides and, on the basolateral side only, replaced with Pneumacult ALI medium (StemCell Technologies, Vancouver, BC, Canada), and differentiation of cells (up to 16–18 days) was performed in the ALI condition.

The collection of bronchial epithelial cells (supported by Fondazione per la Ricerca sulla Fibrosi Cistica through the “Servizio Colture Primarie” Istituto G. Gaslini, Genova, Italy) and their study to investigate the mechanisms of transepithelial ion transport were specifically approved (on 8 July 2018) by the Ethics Committee of the IRCCS Istituto Giannina Gaslini following the guidelines of the Italian Ministry of Health (registration number: ANTECER, 042-09/07/2018). Each patient provided informed consent to the study using a form that was also approved by the Ethics Committee.

### 4.3. MTT Cell Viability Assay

CFBE41o- cells were plated on 96-well plates (20,000 cells per well). After 24 h, cells were treated with different concentrations of test compounds or vehicle alone (DMSO). The following day, the 3-(4,5-dimethylthiazol-2-yl)-2,5-diphenyltetrazolium bromide (MTT) substrate was added to the medium at a final concentration of 0.5 mg/mL and incubated for 1 h at 37 °C. Formazan crystals (proportional to the number of viable cells) are then dissolved by the addition of 20 μL of a pH 4.7 solution containing 20% (*w*:*v*) SDS, 50% (*v*:*v*) *N*,*N*-dimethylformamide, 2% (*v*:*v*) acetic acid, and 25 mM HCl, and quantified by recording the absorbance at 570 nm using an Infinite M200 PRO plate reader (TECAN, Life Sciences). All results were reported as the percentage of vehicle-treated cells.

### 4.4. Cell Lysis and Western Blotting

Cells were washed twice with Phosphate Buffered Saline (PBS) and harvested with ice-cold lysis buffer containing 50 mM Tris–HCl (pH 7.5), 150 mM NaCl, 1% NP-40 (*v*/*v*), supplemented with protease inhibitor cocktail (Calbiochem, San Diego, CA, USA) and phosphatases inhibitors cocktail 2 and 3 (Sigma-Merck, St. Louis, MO, USA). Cell lysates were centrifuged at 10,000× *g* for 10 min at 4 °C and protein concentration was determined by the Bradford method. A total of 20 µg of total protein extracts were loaded on SDS-PAGE, blotted on Immobilon-P membranes (Millipore, Burlington, MA, USA), and processed by western blot with the indicated antibodies. Immunostained bands were detected by chemiluminescence with ImageQuant LAS 500 (GE Healthcare Life Sciences, Piscataway, NJ, USA) and quantified with Carestream Molecular Imaging software (Carestream, Rochester, NY, USA).

### 4.5. Cell Transfection and RNA Interference

Plasmid transfections in CFBE41o- cells were performed as in [26]. For RNA interference, CFBE41o- expressing F508del-CFTR were transiently transfected for 48 h with 5 nM of UBC9 (#s599 and #s600) siRNA or scrambled siRNA (#4390843) (Silencer Select siRNAs, Thermo Fisher Scientific) with RNAiMAX (Thermo Fisher Scientific, Waltham, MA, USA), according to the manufacturer’s instructions.

### 4.6. CFTR Stability

F508del-CFTR expressing CFBE41o- cells were treated with DMSO or TAK-981 in combination or not with correctors (10 μM VX-661 + 3 μM VX-445) for 24 h. Subsequently, protein synthesis was inhibited by adding 100 µg/mL CHX to the culture medium, and then cells were harvested at 0, 2, 4, and 8 h post-CHX treatment. Cell lysates were subjected to SDS-PAGE followed by western blotting to evaluate CFTR expression, as described previously.

### 4.7. qRT-PCR

CFBE41o- cells stably expressing the F508del-CFTR were seeded at 3 × 10^5^ cells/well in a 6-well plate and let adhere for 24 h. Next, cells were left untreated or stimulated with TAK-981 for 18 h. Total mRNA was isolated with a “Total RNA Purification kit” (Norgen Biotek Corp., Thorold, ON, Canada) and reverse-transcribed using SuperScript III reverse transcriptase (Thermo Fisher Scientific, Waltham, MA, USA). For quantitative RT-PCR, 5 ng of template was dissolved in a 20 µL solution containing forward and reverse primers (200 nM each) and 10 µL of SensiFAST SYBR No-ROX Mix, 2x (*Bioline*, Randolph, MA, USA). All the reactions were performed in triplicate on a Real-Time PCR Cycler Rotor-Gene 3000 (Corbett Research, Mortlake, Australia); the thermal cycling conditions were as follows: 3 min at 95 °C, 40 cycles at 95 °C 10 s, 58 °C 20 s and 72 °C 30 s. The relative mRNA expression was calculated and expressed as 2^−ΔΔCt^. Primers were as follows: hGAPDH GAAGGTGAAGGTCGGAGT and CATGGGTGGAATCATATTG; hCFTR CTGGGCTAGGGAGAATGATG and GCCTTCCGAGTCAGTTTCAG.

### 4.8. HS-YFP Assay

CFTR activity has been measured using CFBE41o- cells stably expressing both the F508del-CFTR and the halide-sensitive YFP (H148Q/I152L), according to [47] with minor modifications. Briefly, cells were seeded on 96-well black microplates and left to adhere for 24 h. Cells were treated with indicated compounds for a further 18 h, eight replicate wells for each condition. On the day of the experiment, cells were washed with PBS (137 mM NaCl, 2.7 mM KCl, 8.1 mM Na_2_HPO_4_, 1.5 mM KH_2_PO_4_, 1 mM CaCl_2_, and 0.5 mM MgCl_2_) and incubated with 20 µM forskolin and 50 µM genistein in PBS, to fully stimulate F508del-CFTR-CFTR, for 25 min at 37 °C. Next, plates were transferred to an EnVision plate reader (Perkin Elmer, Waltham, MA, USA) to determine CFTR activity: YFP fluorescence (Ex:485/14; Em:535/30) was continuously measured for 15 s, 2 s before, and 13 s after the injection of an iodide-enriched solution (185 mM NaI, 2.7 mM KCl, 8.1 mM Na_2_HPO_4_, 1.5 mM KH_2_PO_4_, 1 mM CaCl_2_, and 0.5 mM MgCl_2_); the final I^−^ concentration was 100 mM.

To determine the I^−^ influx rate and CFTR activity, background fluorescence was subtracted and fluorescence was normalized to the initial value. Background fluorescence has been measured before the beginning of the assay. In the same plate used for the assay, background fluorescence was continuously measured for 15 s in at least 6 wells in which cells had not been seeded but filled with the same buffers used in the experiment (PBS and iodide-enriched solution).

The final 12 s of the data recording for each well was fitted with an exponential function to calculate the initial slope (d*F*/d*t*).

### 4.9. Short-Circuit Current Recordings

Snapwell inserts carrying differentiated bronchial epithelia were mounted in a vertical diffusion chamber resembling a Ussing chamber and having internal fluid circulation. Apical and basolateral hemichambers were filled with a symmetrical solution containing (in mM): 126 NaCl, 0.38 KH_2_PO_4_, 2.13 K_2_HPO_4_, 1 MgSO_4_, 1 CaCl_2_, 24 NaHCO_3_, and 10 glucose. Hemichambers were continuously bubbled with a gas mixture containing 5% CO_2_—95% air and the temperature of the solution was kept at 37 °C. The transepithelial voltage was short-circuited with a voltage-clamp (DVC-1000, World Precision Instruments; VCC MC8 Physiologic Instruments, San Diego, CA, USA) connected to the apical and basolateral chambers via Ag/AgCl electrodes and agar bridges (1 M KCl in 2% agar). The offset between voltage electrodes and the fluid resistance was adjusted to compensate for parameters before experiments. The short-circuit current was recorded by analog-to-digital conversion on a personal computer.

### 4.10. Statistical Analysis

Results are presented as mean ± SD. The statistical significance was calculated using the Mann–Whitney test or, in the case of multiple comparisons, by Kruskal–Wallis test, followed by Dunn’s multiple comparison test. Differences were considered statistically significant with *p* < 0.05. All statistical analyses and graphs were produced using Prism (GraphPad 4.0 Software) software.

## Data Availability

The original contributions presented in the study are included in the article/supplementary material, further inquiries can be directed to the corresponding author/s.

## Supplementary Materials

The following supporting information can be downloaded at: https://www.mdpi.com/article/10.3390/ijms25042302/s1.
