# Supplementary material for: SUMOylation Inhibition Enhances Protein Transcription under CMV Promoter: A Lesson from a Study with the F508del-CFTR Mutant
Source: Int J Mol Sci. 2024 Feb 15;25(4):2302. doi: 10.3390/ijms25042302 (PMC10889535; doi:10.3390/ijms25042302)
Supplement: Supplementary file 1 [file ijms-25-02302-s001.zip › ijms-2797949-supplementary.pdf]

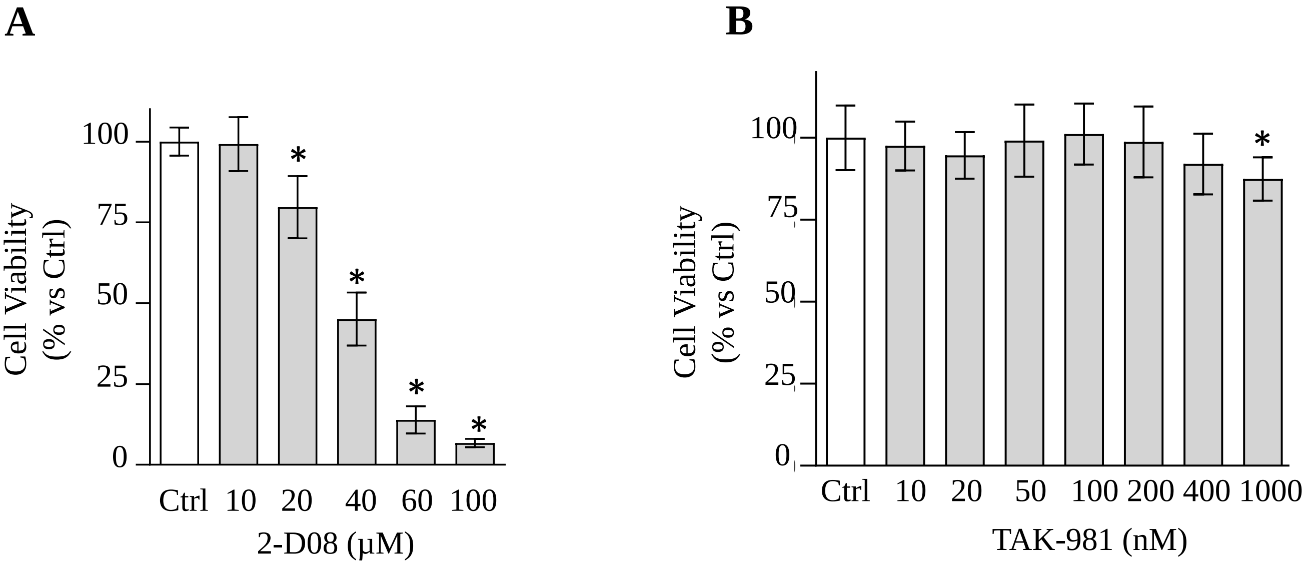

Figure S1. Cell viability of CFBE cells after 48 hours of treatment with 2-D08 or TAK-981. Viability of F508del-CFTR expressing CFBE41o- cells was assessed using the MTT assay after 48 hours of treatment with DMSO (Ctrl) or increasing concentrations of 2-D08 **(A)** or TAK-981 **(B)**. The results were expressed as a percentage of control (Ctrl) (means ± SD values, n = 3; $^*p < 0.05$ vs Ctrl).

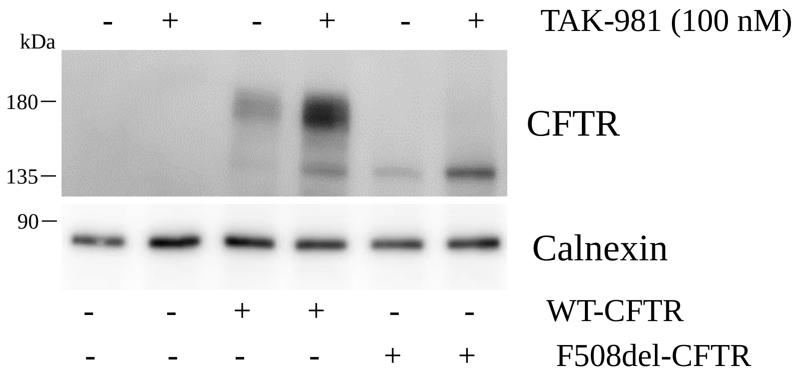

Figure S2. Effect of TAK-981 on transiently expressed WT- and F508del-CFTR in CFBE41o- cells.

CFBE41o- cells were transfected with a plasmid expressing either WT-CFTR or F508del-CFTR. After 24 hours, the cells were treated with DMSO or 100 nM TAK-981 for 24 hours. CFTR expression was analyzed by immunoblotting, with calnexin as the loading control.

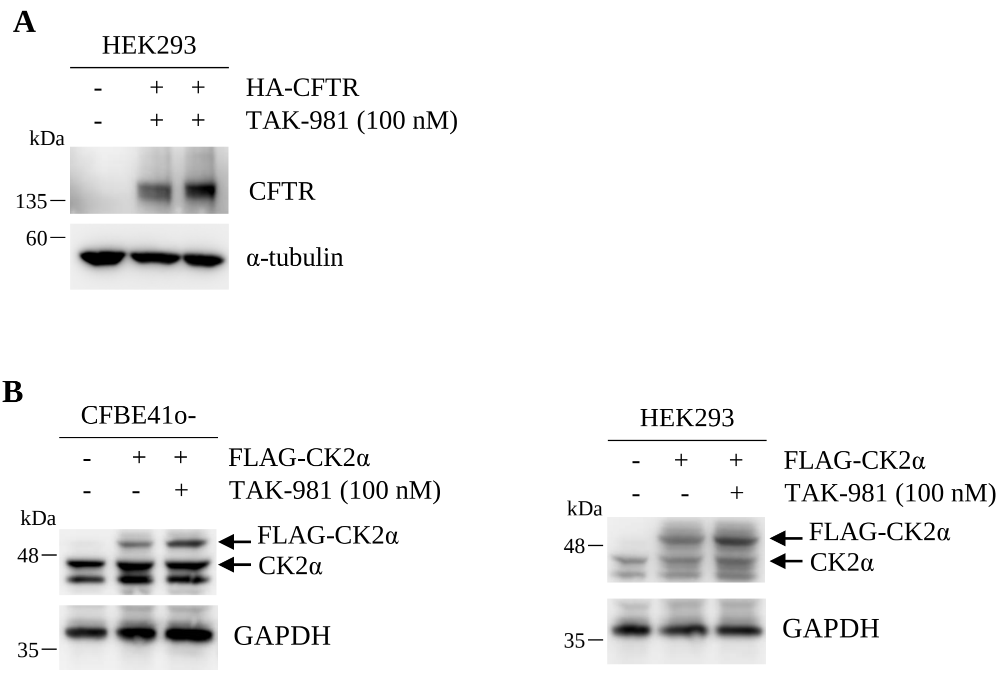

Figure S3. Effect of TAK-981 on transiently expressed proteins in CFBE41o- and HEK293 cells.

(A) HEK293 cells were transfected with a plasmid expressing WT-CFTR. After 24 hours, the cells were treated with 100 nM TAK-981 for 24 hours. CFTR expression was analyzed by immunoblotting, with α-tubulin as the loading control. **(B)** CFBE41o- or HEK293 cells were transfected with a plasmid expressing FLAG-CK2α. After 24 hours, the cells were treated with 100 nM TAK-981 for 24 hours. CK2α expression was analyzed by immunoblotting, with GAPDH as the loading control.

The CK2α antibody identified both the endogenous CK2α (~45 kDa, upper band) and the endogenous CK2α' (~42 kDa, lower band). The FLAG-CK2α band migrated slower than the endogenous protein due to the presence of the tag (FLAG) and is recognized as a separate third band.
